# Moving up the automation S-curve: The role of the laboratory automation support function in successful pharmaceutical R&D

**DOI:** 10.1155/S1463924695000083

**Published:** 1995

**Authors:** Randy J. Maddux

**Affiliations:** Research and Development Services Department Glaxo Inc. Five Moore Drive Research Triangle Park NC 27709 USA

## Abstract

The political and economic climate that exists today is a challenging one for the pharmaceutical industry. To effectively compete in today's marketplace, companies must discover and develop truly innovative medicines. The R&D organizations within these companies are under increasing pressure to hold down costs while accomplishing this mission. In this environment of level head count and operating budgets, it is imperative that laboratory management uses resources in the most effective, efficient ways possible. Investment in laboratory automation is a proven tool for doing just that.

This paper looks at the strategy and tactics behind the formation and evolution of a central automation/laboratory technology support function at the Glaxo Research Institute. Staffing of the function is explained, along with operating strategy and alignment with the scientific client base. Using the S-curve model of technological progress, both the realized and potential impact on successful R&D automation and laboratory technology development are assessed.


Journal of Automatic Chemistry, Vol. 17, No. 2 (March-April 1995), pp. 51-54

### Moving up the automation S-curve: The role of the laboratory automation support function in successful pharmaceutical R&D

**Randy J. Maddux**
*Research and Development Services Department, Glaxo Inc., Five Moore Drive, Research Triangle Park, NC 27709, USA*

*The political and economic climate that exists today is a challenging one for the pharmaceutical industry. To effectively compete in today's marketplace, companies must discover and develop truly innovative medicines. The R&D organizations within these companies are under increasing pressure to hold down costs while accomplishing this mission. In this environment of level head count and operating budgets, it is imperative that laboratory management uses resources in the most effective, efficient ways possible. Investment in laboratory automation is a proven tool for doing just that.*

*This paper looks at the strategy and tactics behind the formation and evolution of a central automation/ laboratory technology support function at the Glaxo Research Institute. Staffing of the function is explained, along with operating strategy and alignment with the scientific client base. Using the S-curve model of technological progress, both the realized and potential impact on successful R&D automation and laboratory technology development are assessed.*

### Introduction

These are challenging times in the pharmaceutical industry. The current debate over the rising cost of health care taking place in Washington, D.C. and around the globe has made the industry a key target for cost reduction initiatives. Glaxo has maintained a highly proactive strategy to discover and develop innovative medicines, while realizing that the value of such medicines must be demonstrated in a concrete manner. The customer base is rapidly changing from the individual physician to a managed care environment where price must be justified. Coupled with this innovation strategy is a concerted effort to find ways to reduce R&D costs and increase speed to market by utilizing resources more effectively and efficiently.

Laboratory managers have long realized that the effective utilization of scientific staff meant freeing them from repetitive, boring tasks and funneling their energies toward more creative activities. Key to this has been the utilization of modern laboratory automation and robotics. Full implementation of the automation strategy, while successful in many companies, often falters due to the lack of necessary technical support in areas where most scientists have little or no training or experience. This is where the internal automation/laboratory technology support function, such as the Bioengineering group within the Glaxo Research Institute (GRI), plays a key role.

This paper was presented at the 1994 ISLAR meeting.

### The S-curve model

The S-curve model [1] (figure 1) is a simple yet effective tool for charting technological progress. The horizontal axis represents resources such as time, money, and staffing levels while the vertical axis represents the performance achieved by allocating these resources. Performance may be measured in terms such as reduction in testing costs, laboratory throughput, or re-deployment of staff, depending on the particular area of interest. Using this model, it is possible to graphically represent important segments of the automation development process such as learning, the point of diminishing returns, and the emergence of discontinuities. Discontinuities occur when the old technology has been fully optimized and the progressive laboratory must look to emerging technologies to continue improving its operating performance.

In addition to serving as a charting tool to track progress, the S-curve may be used as a forecasting tool that allows the laboratory to proactively manage its decisions to invest in new technologies. Proactive decisions in the automation/ laboratory technology arena give the laboratory the ability to maintain the productivity afforded by existing systems while allocating a reasonable portion of its resources to developing new ones.

It is obvious from the shape of the S-curve that the goal of the laboratory should be to minimize the length of the lower and upper flat portions of the curve and to maximize both the slope and length of the middle portion. A fully effective automation/laboratory technology support function such as GRI Bioengineering is key to achieving this overall goal.

### Discussion

The GRI Bioengineering function can trace its origin to a similar function which has existed within Glaxo Research and Development (GRD) in the UK for more than 20 years. The Bioengineering function exists to support laboratory operations in the areas of automation, laboratory technology, and instrument control and support. The name *Bioengineering* is derived from the Biological Engineering Society, a professional organization in the UK and was adopted in the USA for the sake of continuity and because it gave the function a sense of history.

The organizational placement of the Bioengineering function within GRI (figure 2) is critical to its function. Though having formal reporting responsibility to the R&D Services department within GRI Development, the function maintains the necessary autonomy to function as

© Zymark Corporation 1995

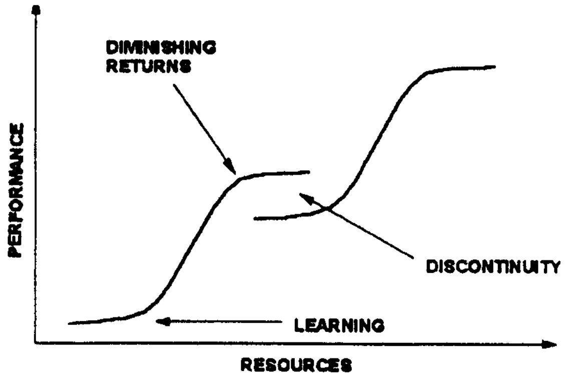

*Figure 1. The S-curve.*

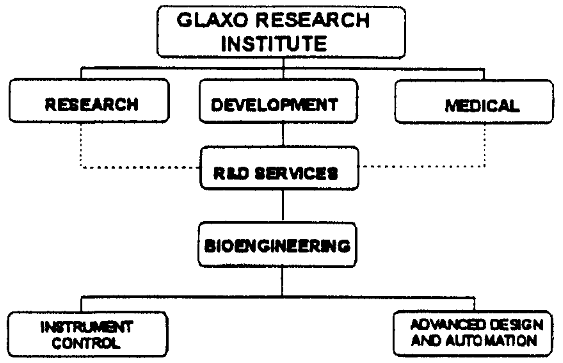

*Figure 2. Organization of GRI.*

a true Glaxo resource, fully supporting the Research and Medical divisions of GRI and providing support to divisions outside GRI as well. It is only through this scope of responsibility that the staffing and resources required to achieve 'critical mass' can be fully justified and effectively utilized.

*The past*

The GRI Bioengineering function began in 1990 as a four member group consisting of a supervisor, two engineers, and a design machinist. Facilities were minimal and project management, for all practical purposes, was nonexistent. Even at this early stage Bioengineering had a positive effect on productivity in the laboratories. For the first time, researchers had an internal resource to support their non routine instrument needs. The success of the group and the value it added to the GRI mission was limited, however, by the fact that few people knew about and were utilizing the group and those who did were hesitant to share this newly discovered resource. GRI Bioengineering was, for all intents and purposes, a 'servant' organization as depicted in figure 3. As a servant organization, the value added by the function was limited to the projects brought forth by the scientist customers. There was no initiative on the part of the function to investigate and bring forth new technologies. Innovation remained the sole responsibility of the customer. Yet, as stagnant as the GRI Bioengineering function was technologically, the real danger to the long term success of the function lay in the lack of systems for project management and documentation. Without supporting

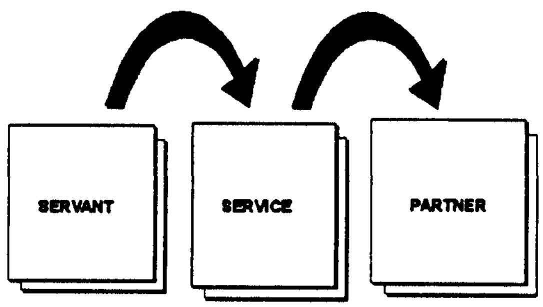

*Figure 3. Evolution of the function.*

evidence, the case for continued and expanded resources was seriously lacking.

*The present*

In 1992, the GRI Bioengineering function began taking on a new look and a new focus. First, the function was split into two separate groups: Instrument Control and Advanced Design and Automation. It was recognized that to fully support the laboratory technology needs of an R&D organization as complex as GRI, separate specialized functions were needed. Instrument Control has as its mission the comprehensive management of laboratory instrumentation and related systems including service and maintenance, vendor contracts, and calibration. The Advanced Design and Automation section is charged with designing, developing, and prototyping novel instruments and automated systems to support innovative R&D initiatives. With a total staff of 15 people and state-of-the-art mechanical design, electronic design, and metrology laboratories, these two sections are no longer merely servant organizations but highly proactive technical service organizations (figure 3) that formulate innovative solutions to highly complex scientific problems. Formal project and data management has been implemented through the use of advanced software tools providing critical justification for resources and maintaining documentation needed for project management and regulatory purposes. The GRI Bioengineering function now actively pursues and implements new technologies as needed to support the GRI scientific community.

*The future*

The key to the future for the GRI Bioengineering function is to make the final leap from service organization to full scientific partner as depicted in figure 3. The effectiveness of the function is optimized under this relationship because no longer is GRI Bioengineering in a reactive or semi-reactive mode of operation but is almost entirely proactive. At the partnership level, strategies and ideas are openly shared between the laboratory technology support group and the client laboratory. This dialogue is conducive to the development of expedient and innovative solutions to even highly complex problems.

The key challenges facing GRI Bioengineering lie both outside and inside of the organization. Laboratory groups may be reluctant to share sensitive technical problems with internal groups outside their immediate organizations and GRI Bioengineering staff must develop good customer interaction and consulting skills in addition to their recognized technical skills.

Being involved as a full partner with its GRI customer base at the strategic and tactical planning level will allow GRI Bioengineering to more fully meet and exceed the needs of its clients on a regular basis.

### Critical success factors

Of the many factors that in one way or another impact the success of the laboratory technology and automation support function, four have been identified as being absolutely critical. These include management commitment and support, organization and staffing, operating strategy and tactics, and marketing of services. As detailed below, each of these factors plays an important role in shaping the function and directly determine its value to the organization.

### *Management commitment and support*

Management commitment and support is important to the success of any function. In the case of the laboratory technology and automation support function, this support is especially critical to its success. The pharmaceutical R&D organization centres its activities around the discovery and development of specific compounds identified as having therapeutic value. The laboratory technology and automation support function must compete with the laboratory functions directly involved with these compounds for available, finite resources. To be successful, the laboratory technology and automation support function is dependent on management that thinks strategically and takes the long view. Rarely, if ever, are the benefits of such a function immediate. It is only over time that the true benefits are realized through the development of innovative instruments and systems that save time and money and facilitate novel experimentation by the scientific staff. It is easy to be overlooked in the battle for resources so the laboratory technology and automation support function must remain a visible and technologically proactive organization. Documentation of successes and effective utilization of resources through sound project management are key to maintaining strong management commitment and support.

### *Organization and staffing*

The organization and staffing of the laboratory technology and automation support function must be in complete alignment with its strategic objectives. To be fully effective, the function must incorporate a variety of skill sets (figure 4) acting together as a team. Staffing should incorporate the scientific and engineering disciplines and the skilled trades, such as design machinists and draftsmen. It is difficult to describe a major system development project that does not have significant mechanical and electronic design components. The scientific components must be added to this to ensure that the resulting system will function in the laboratory, and, of course, someone must actually make non-commercially available parts and assemble the final system.

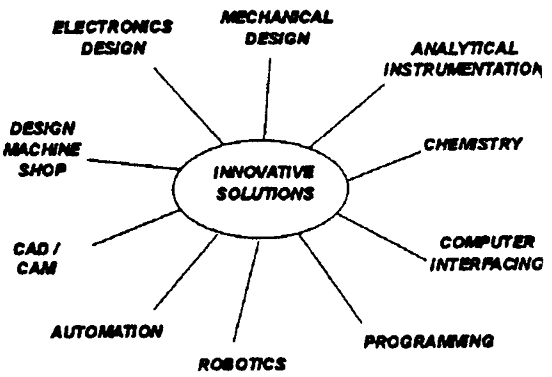

*Figure 4. Staffing—necessary skills mix.*

### *Operating strategy and tactics*

A clearly defined, easily understood operating strategy must be formulated and adhered to by the successful laboratory technology and automation support function. Within GRI Bioengineering this strategy takes the form of a mission statement. The Advanced Design and Automation section has the following mission statement:

> *Our mission is to support innovative R&D by designing, building, or recommending novel scientific devices and equipment based upon state-of-the-art methods and technologies to solve problems and automate processes in the laboratory. Our primary focus is on prototyping new devices and systems which are not commercially available.*

Of course the real value of any mission statement is how well the organization actually adheres to it. It is critical that the organization avoid falling victim to the 'not invented here' syndrome and end up making devices and components that are commercially available. In fact, finding and recommending commercially available solutions is one of the most important ways that the laboratory technology and automation support function adds value to the R&D organization. Constant benchmarking against vendors and peer companies is important to ensure the competitive value of the function. Tactically, it is critical that the laboratory technology and automation support function manage its projects in such a way that customer expectations are met (figure 5). Put simply, '*Establish realistic timelines and meet them!*' It has been the experience of the GRI Bioengineering function that projects extending greater than three months often become irrelevant. It is important to know the limits of the function and employ strategic outsourcing as needed to bring projects to completion in a timely manner.

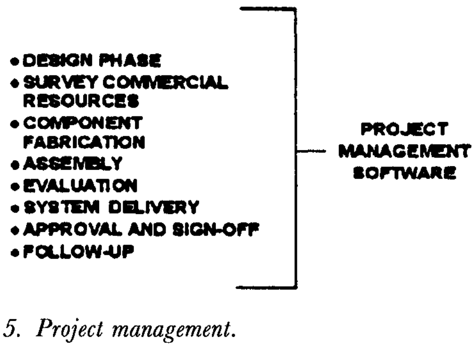

*Figure 5. Project management.*

Finally, it is important to note the word *prototyping* in the Advanced Design and Automation mission statement. A fully developed and tested prototype should trigger efforts to outsource the production model of a particular device or system. With full drawings and specifications available, it is a relatively simple matter to accomplish this, leaving the laboratory technology and automation support function free to focus on the next problem.

### *Marketing of services*

The concept of *marketing* is not often associated with the laboratory technology and automation support function, but it is critical to its long-term effectiveness and ultimate success. Marketing in this sense means making the scientific client base aware of the function and its capabilities. Doing so ensures that the function will have broad exposure to problems across the R & D organization that may determine technology and accompanying staff development initiatives. The overall goal of the marketing effort is to increase the *quality* and strategic importance of the projects taken on, not the *quantity* of work performed.

### Applying the S-curve model

The S-curve model is an effective tool for tracking and forecasting technological progress. The model is readily applied to the area of laboratory robotics, as shown in figure 6. In this example, key areas of the S-curve depict stages in the life of a newly purchased laboratory robot. The first stage, when the robot is purchased, represents learning. During this stage, resources are expended both to purchase the robot and to train its operator(s) with little performance gain to the laboratory. The second stage, when modifications are made and custom modules are built, represents maximum return on investment by the laboratory. During this stage each dollar spent results in significant gains in performance. The third stage represents a fully optimized system and further expenditure of resources results in diminishing gains in performance. As the upper portion of the S-curve flattens, the robot becomes a legacy system and has reached its technological potential. It is at this point that a discontinuity becomes apparent between the existing technology and emerging technology. In the case of robotic systems, this emerging technology may take the form of vision systems, artificial intelligence, or advanced electronics such as fuzzy logic, depending on the particular application. In any case, it is important that the laboratory recognize this discontinuity and manage its resources in such a manner that the emerging technology can be exploited.

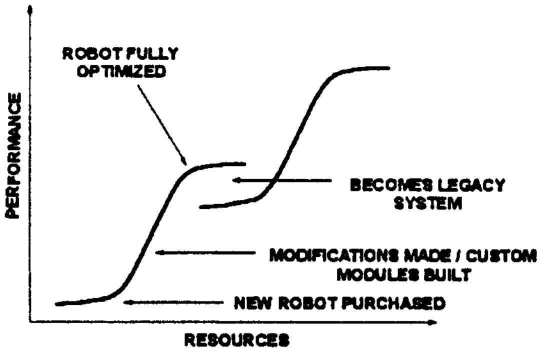

*Figure 6. Applying the S-curve model.*

The laboratory technology and automation support function is an important resource for helping the laboratory chart its course along the S-curve. Through its design and prototyping capabilities, the function may facilitate the move up the steep portion of the curve while its knowledge of state-of-the-art technologies can help the laboratory anticipate and effectively manage the discontinuities.

### Conclusion

This paper has detailed the stragegy and tactics behind the formation and evolution of a central laboratory technology and support function at the Glaxo Research Institute. The S-curve model was demonstrated to be an effective tool for tracking and forecasting technological advancement. If there is a weakness in this model, it is that the model is deceptively simple. Moving up the S-curve requires sound strategic planning and plenty of hard work. The laboratory technology and support function, when optimally organized and managed, can be a key facilitator in this process.

### Acknowledgments

The author wishes to thank Dr Brisco Harward, Manager of Bioengineering Advanced Design and Automation, for his assistance with the production of this paper.

### References

1. Foster, R. N., *Innovation: The Attacker's Advantage* (1986), p. 87.

